# Concentrations of Tetrodotoxin (TTX) and Its Analogue 4,9-Anhydro TTX in Different Tissues of the Silver-Cheeked Pufferfish (*Lagocephalus sceleratus*, Gmelin, 1789) Caught in the South-Eastern Mediterranean Sea, Lebanon

**DOI:** 10.3390/toxins14020123

**Published:** 2022-02-08

**Authors:** Abed El Rahman Hassoun, Ivana Ujević, Sharif Jemaa, Romana Roje-Busatto, Céline Mahfouz, Milad Fakhri, Nikša Nazlić

**Affiliations:** 1 National Council for Scientific Research, National Center for Marine Sciences, Batroun P.O. Box 534, Lebanon; sharif.jemaa@cnrs.edu.lb (S.J.); celine.mahfouz@cnrs.edu.lb (C.M.); milosman@cnrs.edu.lb (M.F.); 2 Laboratory of Plankton and Shellfish Toxicity, Institute of Oceanography and Fisheries, Šetalište Ivana Meštrovića 63, 21000 Split, Croatia; ujevic@izor.hr (I.U.); rroje@izor.hr (R.R.-B.); nazlic@izor.hr (N.N.)

**Keywords:** marine toxins, seafood security, pufferfish, invasive species, Mediterranean Sea, Lebanon

## Abstract

Pufferfishes are among the best-known marine organisms that accumulate marine biotoxins such as Tetrodotoxin (TTX). In the Mediterranean Sea, the silver-cheeked toadfish *Lagocephalus sceleratus* is the most reported TTX-bearer, causing many fatal and non-fatal cases. In Lebanon, no previous studies have measured TTX levels although the possibility of TTX-poisoning is high since *L. sceleratus* is caught in different sizes and can be mistaken with other small fishes. Hence, this study reports TTX and its analogue 4,9-anhydro TTX in *L. sceleratus* collected from Lebanese waters in the Eastern Mediterranean Sea. The results show that TTX concentrations in fish tissues varied between 0.10 and 252.97 µg/g, while those of 4,9-anhydro TTX oscillated between 0.01 and 43.01 µg/g. Internal organs of *L. sceleratus* were the most toxic parts of its body, with the highest TTX levels found in gonads (mainly ovaries) and liver, followed by the muscles and skin with concentrations always exceeding the safety level. Toxicity fluctuations of *L. sceleratus*, its expansion, ecological and economic effects were also elucidated. Based on the present findings, it has been confirmed that *L. sceleratus* constitutes a health, ecological and economic risks, and therefore its trade in seafood markets should be banned to avoid any potential intoxication.

## 1. Introduction

Marine biotoxins are naturally existing chemicals produced by toxic algae that can accumulate in marine biota [1]. However, some biotoxins are thought to be produced primarily by marine bacteria. These toxins may bioaccumulate through the food chain or in combination with parasitism or symbiosis (a part of the animal’s natural microbiome), thus intoxicating detritus feeders, small carnivores, omnivores/scavengers, and organisms higher up the food chain [2]. This is the case of Tetrodotoxin (TTX), a non-protein, weakly basic organic, and low molecular weight compound that is soluble in water and acidic environments, colorless, odorless, tasteless, and heat resistant [3,4,5]. Approximately 25 naturally occurring analogues of TTX have been detected and many of these have also been shown to have toxicity potential [6]. TTX is as much as 1200 times stronger than the well-known poison cyanide [6]. It acts by binding and thus blocking the voltage-gated sodium channels of the musculatory and nervous system [7,8]. Although TTX has been detected in a variety of marine animals [9], 93.7% of intoxications are due to fishes of the order Tetraodontiformes, namely pufferfish, that are the most-known fish genus containing this potent neurotoxin [10]. In fact, pufferfish might be attracted by TTX and select TTX-bearing organisms as food, resulting in accumulation of TTX as a possible biological defense agent [2]. Due to the presence of high TTX levels in its various tissues, *Lagocephalus sceleratus* (Gmelin, 1789) is considered one of the most poisonous fish in the world. This explains the high number of studies related to TTX occurrence in the Mediterranean pufferfish, particularly in this fish species [4,5,11,12,13,14,15].

### 1.1. Lagocephalus sceleratus (Gmelin, 1789)

The marine fish species *Lagocephalus sceleratus* (Gmelin, 1789), also known as the silver-cheeked toadfish, belongs to Tetraodontidae family that was introduced in the Mediterranean via the “Lessepsian migration”. This latter term was invented to characterize the unidirectional migration of the Indo-Pacific/Red Sea marine species that have crossed the Suez Canal since 1869 towards the Eastern Mediterranean Sea [16], where they have established successful settlements, thus becoming a major component of the Mediterranean Sea marine biota [12,17,18]. More than 300 Lessepsian species from various taxa were brought to the Mediterranean Sea and *L. sceleratus* is considered among the most dangerous in terms of toxicological, ecological and economic impacts [7,11,16,19]. *L. sceleratus* has been included in the blacklist as one of the 18 worst invader fish species by the IUCN [20]. This species is mostly found between 18 and 100 m depth [21] and does not have predators in the Mediterranean Sea [22]. In addition, it can inflate itself with water and air as a defense mechanism which makes it more difficult to be swallowed by potential predators [23], which additionally explains the exponential increase in its number [7,22]. Based on the available literature, the first record of *L. sceleratus* in the Mediterranean Sea dates back to February 2003 in Gökova Bay, located in the Southern Aegean Sea, Turkey [24]. However, *L. sceleratus* was reported for the first time in Lebanese waters during 1977 [25], showing that this species has been expanding in the Levantine Sub-basin of the Mediterranean Sea long before the first observations became available in the literature in 2003 and subsequently in 2004 for the South-Eastern Levantine Sea [26,27]. Afterwards, the expansion of this species was recorded in various Mediterranean sub-basins, confirming its ability to adapt to different environments [7,28].

### 1.2. TTX Intoxication

Food poisoning related to the consumption of TTX-bearing organisms, such as pufferfish, occurs when the flesh and/or organs of the fish are improperly prepared and eaten. There are no known antidotes or antitoxins to treat TTX-intoxicated patients [5,29]. The number of TTX intoxications has been increasing worldwide with reports from Australia, Bangladesh, Brazil, China, India, Japan, Madagascar, Reunion Island, Spain, Taiwan, Thailand, the United States, and Tanzania [2,8,30,31,32,33,34,35,36,37,38,39,40]. Compared to the rest of the world, around 38% of TTX-related fatalities occurred in Europe and the Middle Eastern/African countries (21.4 and 16.7%, respectively) [10]. In the Mediterranean Sea region, due to its ‘famous’ toxicity, the human consumption of *L. sceleratus* is not common. In addition, according to the European legislative requirements [41,42], poisonous fish of the family Tetraodontidae and derived products must not be placed on the market. However, accidental capture and consumption of this species has been documented in many parts of the Eastern Mediterranean Sea [11,29,43]. Depending on the amount of the ingested TTX, symptoms might initiate with oral paresthesia that spreads to the extremities and body with disturbance, dizziness, headache, and diaphoresis, and can impact the gastrointestinal and/or nervous and mobility systems causing paralysis, muscle coordination disorders, respiratory depression, circulatory failure, and nausea, and eventually death [11,29,44,45,46,47,48,49]. Symptoms usually appear within 10 to 45 min, and exceptionally 3 to 6 h after exposure [11].

All the above-mentioned serious health issues, which may result from accidental consumption of TTX-bearing organisms such as *L. sceleratus*, show that TTXs can now be considered among the most threatening emerging toxins to food security in Europe. Nevertheless, maximum permitted limits have not yet been set, and no specific monitoring plans have been adopted so far. This emphasizes the importance of measuring its concentrations in species from around the Mediterranean, such as the Lebanese waters, to test its toxicity. A TTX-poisoning case was documented in Lebanon, with serious symptoms that occurred within 3 to 4 h after the consumption of *L. sceleratus* gonads [29], although there are regulations that prohibit the selling of pufferfish in Lebanese seafood markets (No. 676/1 of 27 July 2011). Despite this incident, there is no TTX reporting yet to highlight the toxicity of pufferfish based on scientific data collected from local species. Therefore, this study constitutes the first report of TTX concentrations in various tissues of *L. sceleratus* collected from the Lebanese waters in the South-Eastern Mediterranean Sea. The presented results provide information on this species in order to raise the public awareness of its toxicity.

## 2. Results

The *L. sceleratus* specimens were sampled at the same location, and details of their morphological and sex analysis, as well as the range of TTX and its analogue concentrations in their different organs and tissues, are shown in Table 1.

### 2.1. TTX Concentrations

**Figure 1:**
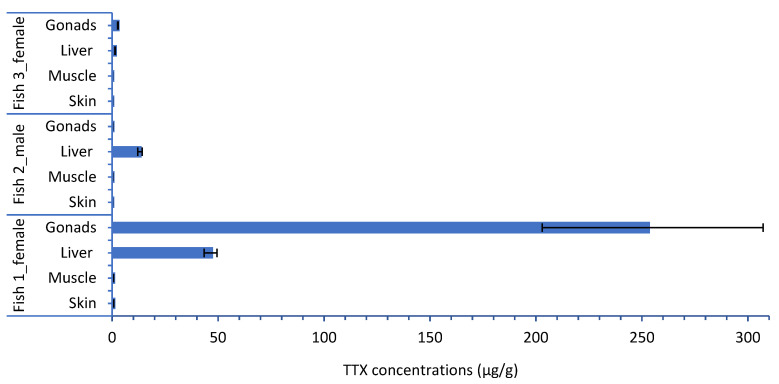
TTX concentrations (μg/g) in organs (gonads, liver, muscle and skin) of each *L. sceleratus* specimen (bars represent minimum and maximum of TTX concentrations, *n* = 5).

**Figure 2:**
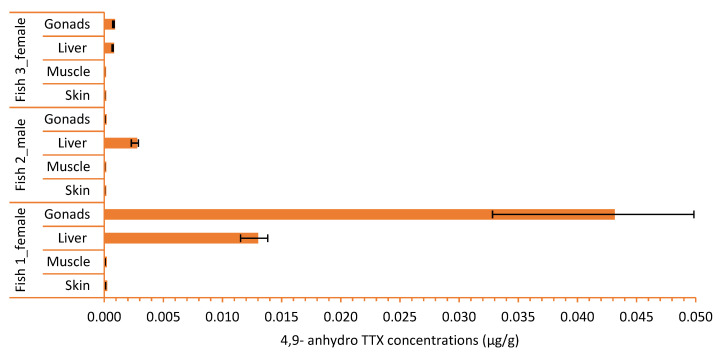
4,9-anhydro TTX concentrations (μg/g) in organs (gonads, liver, muscle and skin) of each *L. sceleratus* specimen (bars represent minimum and maximum of 4,9-anhydro TTX concentrations, *n* = 5).

The results demonstrate that concentrations of TTX and its analogue 4,9-anhydro TTX follow the same pattern in all the fish specimens and their individual organs (Table 1; Figure 1 and Figure 2). The highest TTX and 4,9-anhydro TTX concentrations were detected in Fish 1, the largest and the most mature fish examined (252.97 and 43.01 μg/g for TTX and 4,9-anhydro TTX, respectively); whereas the lowest concentrations were found in Fish 3, the smallest one (0.10 and 0.03 μg/g for TTX and 4,9-anhydro TTX, respectively; Table 1).

Based on the results, it is clear that TTX and 4,9-anhydro TTX concentrations may vary considerably with respect to different organs of *L. sceleratus*, exhibiting the highest concentrations in gonads (in ovaries), then in liver, followed by the muscles and skin (Figure 1 and Figure 2, Table 1), while it is higher in the liver in testis (Table 1).

### 2.2. TTX Toxicity

**Figure 3:**
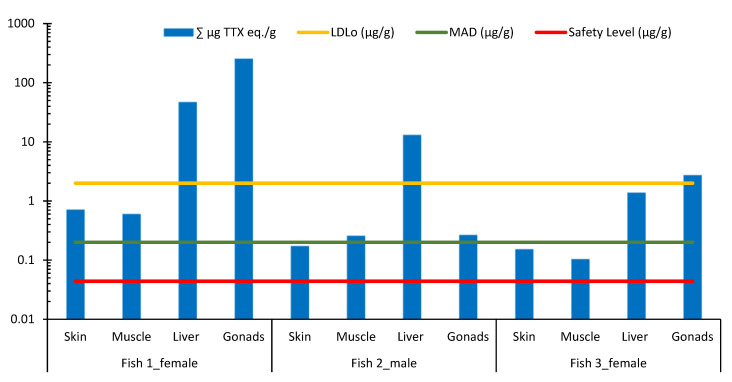
Logarithmic presentation of combined TTX and 4,9-anhydro TTX concentrations (μg/g) in each organ of *L. sceleratus* specimens with visualization of the Lowest Lethal Dose (LDL_0_), the Minimum Acute Dose (MAD), and the Safety level (according to [6]).

All measured TTX and 4,9-anhydro TTX concentrations in all analyzed fish organs exceeded the safety level set by the European Food Safety Authority (EFSA) for toxic effects in humans, which is 0.044 μg/g for TTX and its analogues, based on the consumption of 400 g of marine bivalve and gastropods/shellfish meat [6]. For example, the muscle of the smallest fish (Fish 3) presented a concentration of TTX and 4,9-anhydro TTX combined (0.103 μg/g) more than twofold higher than the determined safety level (Figure 3).

Only the skin and muscle of the smallest Fish 3 showed combined TTX (TTX and 4,9-anhydro TTX) concentrations lower than the Minimum Acute Dose (MAD = 0.2 μg/g) [11]; whereas livers of the largest and medium-sized fish (Fish 1 and 2, respectively) contained concentrations greater than the LDL0. Female gonads (ovaries) of both Fish 1 and Fish 3 comprised concentrations above the Lowest Lethal Dose (LDL0 = 2 μg/g; Figure 3) [11].

## 3. Discussion

### 3.1. TTX Toxicity in Lebanon, the South-Eastern Mediterranean Sea

Despite the fact that pufferfish species, in general, are not consumed in the Mediterranean region, about 156 intoxication cases have been recorded in various Mediterranean countries, and have mainly been attributed to *L. sceleratus*, as shown in a detailed overview by Guardone et al. [10]. This overview revealed that TTX fatality rates vary between 16.7% in Middle Eastern and North African countries and 21.4% in European countries, both constituting ~38% of the world’s TTX-related fatalities. Furthermore, the highest numbers of cases have been noted for the Southern and South-Eastern shores, namely in Middle Eastern and North African countries, where 89.5% of cases were non-fatal and 10.5% were fatal [10]. In the vicinity of the Suez Canal, it is obvious to record the Lessepsian species *L. sceleratus*, that has been recorded there more than 40 years ago [25], and which explains the high number of TTX-poisoning cases in the South-Eastern Mediterranean region [25,27,43,48]. All the mentioned poisoning incidents emphasize the role of *L. sceleratus* as an emerging issue related to food safety and public health in the Mediterranean region [10,50].

In the present study, muscles and skin of *L. sceleratus* in Lebanese waters showed TTX concentrations lower than those measured in the same organs of specimens collected from other parts of the Mediterranean Sea (Cyprus, Greece, Spain and Turkey), as well as the Red Sea and the Indian Ocean (Table 2). However, TTX concentrations measured in the gonads and liver of fish species collected in this study are considered high (Table 2); primarily the gonads that present the highest TTX concentrations based on the findings in the available literature (252.97 μg/g in the largest Fish 1; Figure 3). Results from this study demonstrate that internal fish organs are the most toxic ones, in agreement with previous studies on *L. sceleratus* [2,11,51,52,53]. Moreover, toxicity levels in different fish organs reported in this paper are in line with the ones noted in published studies obtained via diverse methods of analyses (Table 2), as the highest TTX concentrations were found in gonads (mainly ovaries) and liver, followed by muscles and skin. Nonetheless, the toxicity content in muscles reported in this study was much lower than in other ones (<0.7 μg/g), while still exceeding the safety level 0.044 μg/g [6]. This TTX distribution through organs is attributed to the diet of pufferfish that eats food containing this toxin which goes initially to the liver, then to the skin/gonads and other organs, but also to the prolonged degradation of TTX in gonads [7,54]. The median lethal dose (LD50) concentrations for TTX in mammals are 0.01–0.014 μg/g for subcutaneous and 0.002–0.01 μg/g for intravenous administration [55,56]. In the present study, the majority of analyzed organs of the three investigated fish specimens show that TTX concentrations exceed the minimum acute dose of TTX for humans (wt. 50 kg; 0.2 mg; Figure 3), which is a dose that causes adverse health effects [57]. Therefore, the present study, which is in harmony with the above-mentioned ones, clearly shows that *L. sceleratus*, purchased from the seafood market of Lebanon, is a toxic fish species and that even small individuals are not safe for consumption, since all the measured values of TTX in all the analyzed fish organs were above the safety level.

Our results demonstrate the potential toxicity of *L. sceleratus* when consumed, since a couple of accidental intoxication cases related to its consumption were recently reported in Lebanon [29,49]. One of the straightforward reports related to TTX-poisoning in Lebanon describes the effects noted after the consumption of *L. sceleratus* gonads by a Lebanese man who entered a deep non-reactive reversible coma with the absence of brainstem reflexes within 3 to 4 h [29]. The symptoms started with perioral tingling, followed by dysarthria, after which the patient became quadriparetic, then developed respiratory and hemodynamic failure. The patient started to improve after 20 h and recovered his neurological baseline within 36 h. All these symptoms have been attributed to TTX that blocked voltage-gated sodium channels and inhibited the production and propagation of action potentials [29]. Such incidents could be occurring because *L. sceleratus* juveniles are considered to be non-toxic and small individuals can be mistaken for some other edible fish species [58]. Moreover, the catch of the two Tetraodontidae toxic species, *Lagocephalus sceleratus* and *Torquigener flavimaculosus*, in purse seine fisheries in Lebanon were recently reported for the first time. Such new findings highlight the existence of a danger to consumers via accidental poisoning [60], as the presence of these toxic species in fish assemblages increases the potential of intoxication incidences among the consumers who eat small pelagic fish, as they do not distinguish small–juvenile fish species and traditionally eat them without the evisceration [1,60], with their internal organs bearing TTX levels above the LDL0 and MAD (Table 2). This reflects a significant TTX-related risk on consumers, since the treatment is only symptomatic and mostly aggressive with a mortality rate around 60% [61].

Results in this study also demonstrate the relationship between the high toxicity of *L. sceleratus* and the size of fish—the bigger the fish the more toxic it is, which is in agreement with results found in other studies [11]. Although the organ distribution of toxins is reported to be species-specific [62], the geographic location and seasonal variation may influence the toxicity levels as the ecological food chain, the accumulation of the TTX-producer bacteria, local variations of toxicity, and the toxin composition in fish may vary in function of habitats and seasons [2,3,12,51,63,64,65].

Interesting results in the present study were embodied by the high concentrations of TTX in ovaries (female gonads of Fish 1 and 3) compared to the concentrations measured in testis (male gonads of Fish 2). The same pattern of gonads’ toxicity was also noted in other studies on *L. sceleratus* [5], as well as on *Lagocephalus lunaris* and *Chelonodon patoca* where muscles and/or testis were non or weakly toxic [2]. Similar findings have been noted for fish from Cyprus by Akbora et al. [7] who mentioned that in winter, spring, and summer seasons females had higher toxicity levels than males.

The prolongation of this study throughout all the seasons would be interesting to evaluate the seasonal toxicity fluctuations in *L. sceleratus* invading the Lebanese waters, as well as the influence of fish gender on TTX levels.

### 3.2. Expansion of L. sceleratus

*L. sceleratus* is not limited to the Eastern Mediterranean Sea anymore, as it has been recorded in the Northern and Southern realms of the Western Mediterranean [66,67,68,69,70,71,72,73,74], as well as in the westernmost end of the Strait of Gibraltar at San Amaro beach, Spain, which opens the possibility for this Indo-Pacific invasive species to spread beyond the Mediterranean Sea, risking the future expansion of Lessepsian bioinvasions to the Atlantic Ocean [75]. Moreover, the undergoing ecological change attributed to global warming and other climate change-related consequences can be a potential reason behind the expansion of invasive species, such as *L. sceleratus* in the Mediterranean Sea [11,12,76] and pp. 328–374 in [76]. However, more scientific research and risk assessments with regards to its toxicity and other potential public health concerns have to be undertaken.

### 3.3. Regulations

All the above-mentioned findings highlight the need for a new risk-based approach to control TTX-caused food safety issues, but also considering that to this date a maximum permitted TTX concentration in seafood has not been established by the European legislation [6]. Currently, EU regulation only manages the risk by prohibiting the marketing of fishery products obtained from fish of the Tetraodontidae families, Molidae, Diodontidae and Canthigasteridae [41], thus not yet considering other marine product categories that could potentially carry TTX [10]. Moreover, in order to efficiently and rapidly avoid TTX-related intoxications, the availability of low-cost and user-friendly tools for TTXs detection will contribute to guarantee seafood safety [58]. All these regulations and new techniques should be implemented as soon as possible since once *L. sceleratus*, or other TTX-bearers and invasive species, manage to establish stable populations in a new environment, they can hardly be eradicated and therefore urgent action in the form of control of spread and removal are very important, as these are often the only effective methods in combating their harmful effects [15].

In Lebanon, after a couple of human intoxications due to pufferfish consumption, the Ministry of Agriculture (responsible for fisheries) issued a decision (No. 676/1 of 27 July 2011) that prohibits the catchment, transportation, selling, and the consumption of both *Lagocephalus* sp. and *Torquigener* sp.

## 4. Conclusions

In this study, morphologically different individuals of *L. sceleratus* collected from the Lebanese waters were analyzed for TTX and its analogue 4,9-anhydro TTX concentrations. The obtained results demonstrated that TTX concentrations varied between 0.10 and 252.97 µg/g, where internal fish organs were the most toxic ones with TTX showing the highest concentrations in gonads (mainly ovaries) and liver, followed by muscles and skin where measured toxicity always exceeded the safety level (0.044 μg/g) [6]. Another remarkable result was the high concentrations of TTX found in ovaries compared to the ones measured in testis, showing a potential relationship between the toxicity and the gender of fish. Moreover, this paper warns of the toxicity of *L. sceleratus*, and gives insights on its expansion and its ecological and economic effects. Finally, this paper offers a few recommendations to insist on the importance of implementing systematic monitoring of already-known and emerging TTX-bearing organisms in order to avoid any potential human intoxication and to control the expansion of this invasive species.

## 5. Materials and Methods

### 5.1. Samples Collection and Preparation

**Figure 4:**
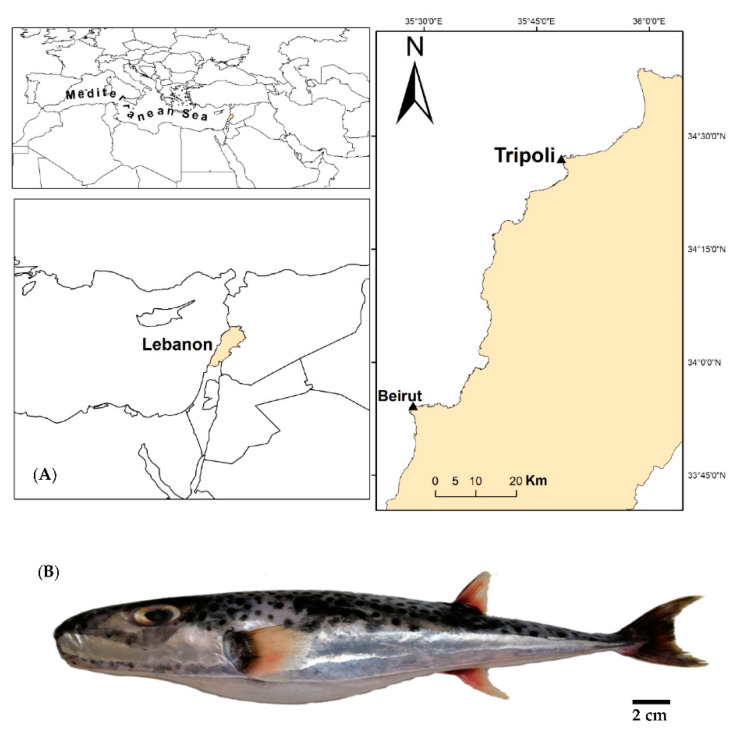
(**A**) Sample collection area; (**B**) a photo of *Lagocephalus sceleratus*© Sharif Jemaa, CNRS-L.

Three specimens of pufferfish *Lagocephalus sceleratus* (Gmelin, 1789) were bought from a local fisherman directly after collection by a gillnet offshore Tripoli city, North Lebanon (Figure 4), in February 2020 (winter), and frozen at −20 °C for one night. Morphological and sex determination assessments of each individual were conducted in order to obtain information on their total length, weight, sex, and maturity. The total length (TL), to the nearest millimeter and total weight (TW), to the nearest 0.01 g of the fish were recorded for each specimen. Sexual maturity was determined using the instruction manual for the MEDITS survey 6 [77]. Each individual was partially thawed in order to avoid toxin cross-contamination between tissues or organs, and subsequently dissected into muscles, liver, skin, and gonads. After species identification, dissection, and morphological measurements, each organ of the fish was weighed, then frozen and transported to the Institute of Oceanography and fisheries in Split (Croatia) for TTX analysis via LC-MS/MS.

### 5.2. Sample Extraction

Three individual fish specimens were dissected and 2 g of each of the following organs were weighed: muscle, liver, gonads, and skin. Extraction started by adding 7.5 mL of 1% acetic acid to polypropylene test tube containing each fish tissue that was homogenized at 2400 rpm for 10 min on Ultra-turrax homogenizer (Cole-Parmer, GEN 700, Vernon Hills, IL, USA), then placed in an ultrasonic water bath for 10 min at 35 kHz (DT 100 H, Bandelin sonorex digitec, Germany) followed by a boiling water bath (100 °C) for 10 min. After cooling to room temperature (25 °C) and volume adjusted to 7.5 mL by adding deionized water, the extracts were centrifuged at 3000× *g* for 20 min at 4 °C (Hettich Zentrifugen, Rotina 420, Germany) and the supernatants were filtered through 0.2 μm nylon syringe filters and poured directly in autosampler glass 2 mL vials for LC-MS/MS analysis.

The Certified Reference Materials (Cifga laboratorio, Lugo, Spain) contained the following concentrations of TTX and its analogue: 25.9 ± 1.3 µg/g TTX and 2.99 ± 0.16 µg/g 4,9-anhydro TTX.

In order to quantitatively determine TTX concentrations, a highly sensitive analytical method was employed using the Agilent mass spectrometer operated in positive ion mode via electrospray ionization (ESI). An Agilent Technologies LC-MS/MS analytical system coupled with Triple Quad 6410, Degasser 1200, Quaternary Pump 1200, Auto Sampler 1290 and Thermostatted Column Compartment 1290 was used, while the LC system was equipped with a Poroshell 120 EC-C18 column (2.1 × 50 mm, 2.7 μm) and Poroshell 120 EC-C18 Guard precolumn (2.1 × 5 mm, 2.7 μm). Water (35%) and 95% acetonitrile (65%), both containing 2 mM ammonium formate and 50 mM formic acid were the mobile phase solvents; the flow rate was set to 0.3 mL/min and the column temperature to 25 °C. Standard solution of TTX and 4,9-anhydro TTX was used to optimize the MS parameters, including the molecular ion peak (M + H)+ occurring at m/z 319.8, which showed the molecular weight for TTX (319.1), and the molecular ion peak (M+H)+ occurring at m/z 302.1, which showed the molecular weight for 4,9-anydro TTX (302.2). Two product ions were analyzed in the spectrometer and the transitions were m/z 319.8/302.0 and m/z 319.8/161.9 for TTX and 302.1/256.0 and 302.1/162.0 for 4,9-anhydro TTX.

Standard calibration curves were obtained by using an externally certified reference solution (Cifga laboratorio, Lugo, Spain) prepared in six different concentrations for TTX (20.0, 50.0, 100.0, 200.0, 500.0, and 1000.0 ng/mL) and for 4,9-anhydro TTX (2.37, 5.94, 11.87, 29.76, 59.63, and 118.73 ng/mL), all measured in triplicate. The detection limits of 1.43 ng/mL and 2.10 ng/mL for TTX and 4,9-anhydro TTX, respectively, were obtained from three Standard Deviations (SD) of seven measurements of the lowest standard, while the limits of quantification of 4.77 ng/mL for TTX and 7.01 ng/mL for 4,9-anhydro TTX were obtained from 10 SD of seven measurements of the lowest standard (see supplementary document to check the calibration plots).

## Figures and Tables

**Table 1:** Morphological details of *L. sceleratus* and TTX and 4,9-anhydro TTX concentration ranges in organs (gonads, liver, muscle, and skin) of each fish specimen (μg/g). Total Length (TL), Total Weight (TW), Liver Weight (LW) and Gonads Weight (GW).

Fish Code	TL (cm)	TW (g)	Sex	Maturity	LW (g)	GW (g)	Range of TTX (μg/g)	Range of 4,9-Anhydro (μg/kg)	TTX/4,9-Anhydro (μg/g)			
									Muscle	Skin	Liver	Gonads
Fish 1	58.9	2608.5	Female	4	245	50.1	0.60–252.97	0.08–43.01	0.60/7.5 × 10^−5^	0.71/11.3 × 10^−4^	46.67/0.013	252.97/0.043
Fish 2	48.3	1238.8	Male	2	58.7	5.4	0.17–12.96	0.03–2.65	0.26/3.2 × 10^−5^	0.17/2.8 × 10^−5^	12.96/0.0026	0.26/5.8 × 10^−5^
Fish 3	19	82.2	Female	2	4	0.8	0.10–2.71	0.03–0.77	0.10/1.2 × 10^−5^	0.15/2.7 × 10^−5^	1.37/6 × 10^−4^	2.71/7 × 10^−4^

**Table 2:** TTX concentrations (µg/g) in muscle, gonads, liver and skin of *L. sceleratus* from different studies, including the present one (in bold).

		Region	Muscle	Gonads	Liver	Skin	Method
Mediterranean Sea	[11]	Rhodes Island, Greece	<1.10–10.1	17.05–239	16.12–88	<1.10–6.63	MBA
	[12]	Rhodes Island, Greece	<LOQ–3.47	0.47–46.3	4.20–44.2	<LOQ–1.40	LC-ESI-CID-MS/MS
	[4]	Mersin Bay, Turkey	ND–2.83	0.43–52.1	ND–46.2	0.13–3.43	MBA & LC-MS/MS
	[14]	Marmaris and Iskenderun Bay, Turkey	0.10–3.42	0.17–80.0	0.12–25.4	0.10–3.30	LC-MS/MS
	[13]	Spain	0.7–0.9	20.0–21.1	2.3–4.6	1.2–1.8	LC-MS/MS & LCHRMS
	[5]	Mersin Bay, Turkey	0.70–5.12	0.69–35.6	0.89–21.1	2.20–11.8	Q-TOF LC/MS
	[58]	Chrousou Bay, Greece	0.48–2.88	-	0.73–10.83 *	1.19–3.17	Electrochemical MB-based immunosensing tool, LC-HRMS and mELISA
	[7]	Northern Cyprus	0.21–8.32	0.32–12.87	0.11–13.48	0.16–6.54	dcELISA
	**Present study**	**Tripoli, Lebanon**	**0.10–0.59**	**0.26–252.97**	**1.37–46.67**	**0.15–0.70**	**LC-MS/MS**
Red Sea	[51]	Suez, Egypt	ND–27.9	ND–165	ND–54.1	ND–26.2	TLC and electrophoresis
Indian Ocean	[3]	Malaysia	1.71	-	-	-	GC-MS
	[38]	Reunion Island	5	-	17	-	MBA
	[59]	Malaysia	30	-	24.7	0.51	LC-MS/MS

* in internal organs of juvenile *L. sceleratus* (liver and intestinal tract). -No data, ND = Not Detected.

## Data Availability

Data can be shared upon request.

## Supplementary Materials

The following supporting information can be downloaded at: https://www.mdpi.com/article/10.3390/toxins14020123/s1, Supplementary Figure S1: Total ion current plots of LC-MS/MS derived by the multiple reaction monitoring (MRM) of (a) TTX standard calibration solution (Retention Time = 0.8 min) and Lagocephalus sceleratus tissue extracts of: (b) skin, (c) muscle, (d) liver and (e) gonad, sampled at the South-Eastern Mediterranean Sea, Lebanon. Supplementary Figure S2: Response of the instrument and concentrations of standard solutions are linear, r^2^ > 0.99, as shown for TTX. Supplementary Figure S3: Response of the instrument and concentrations of standard solutions are linear, r^2^ > 0.99, as shown for 4,9-anhydro TTX.
