# Supplementary material for: Concentrations of Tetrodotoxin (TTX) and Its Analogue 4,9-Anhydro TTX in Different Tissues of the Silver-Cheeked Pufferfish (Lagocephalus sceleratus, Gmelin, 1789) Caught in the South-Eastern Mediterranean Sea, Lebanon
Source: Toxins (Basel). 2022 Feb 8;14(2):123. doi: 10.3390/toxins14020123 (PMC8877804; doi:10.3390/toxins14020123)
Supplement: Supplementary file 1 [file toxins-14-00123-s001.zip › toxins-1448943-supplementary.pdf]

Supplementary Materials: Concentrations of Tetrodotoxin (TTX) and Its Analogue 4,9-Anhydro TTX in Different Tissues of the Silver-Cheeked Pufferfish (*Lagocephalus sceleratus*, Gmelin, 1789) Caught in the South-Eastern Mediterranean Sea, Lebanon

Abed El Rahman Hassoun, Ivana Ujević, Sharif Jemaa, Romana Roje-Busatto, Céline Mahfouz, Milad Fakhri and Nikša Nazlić

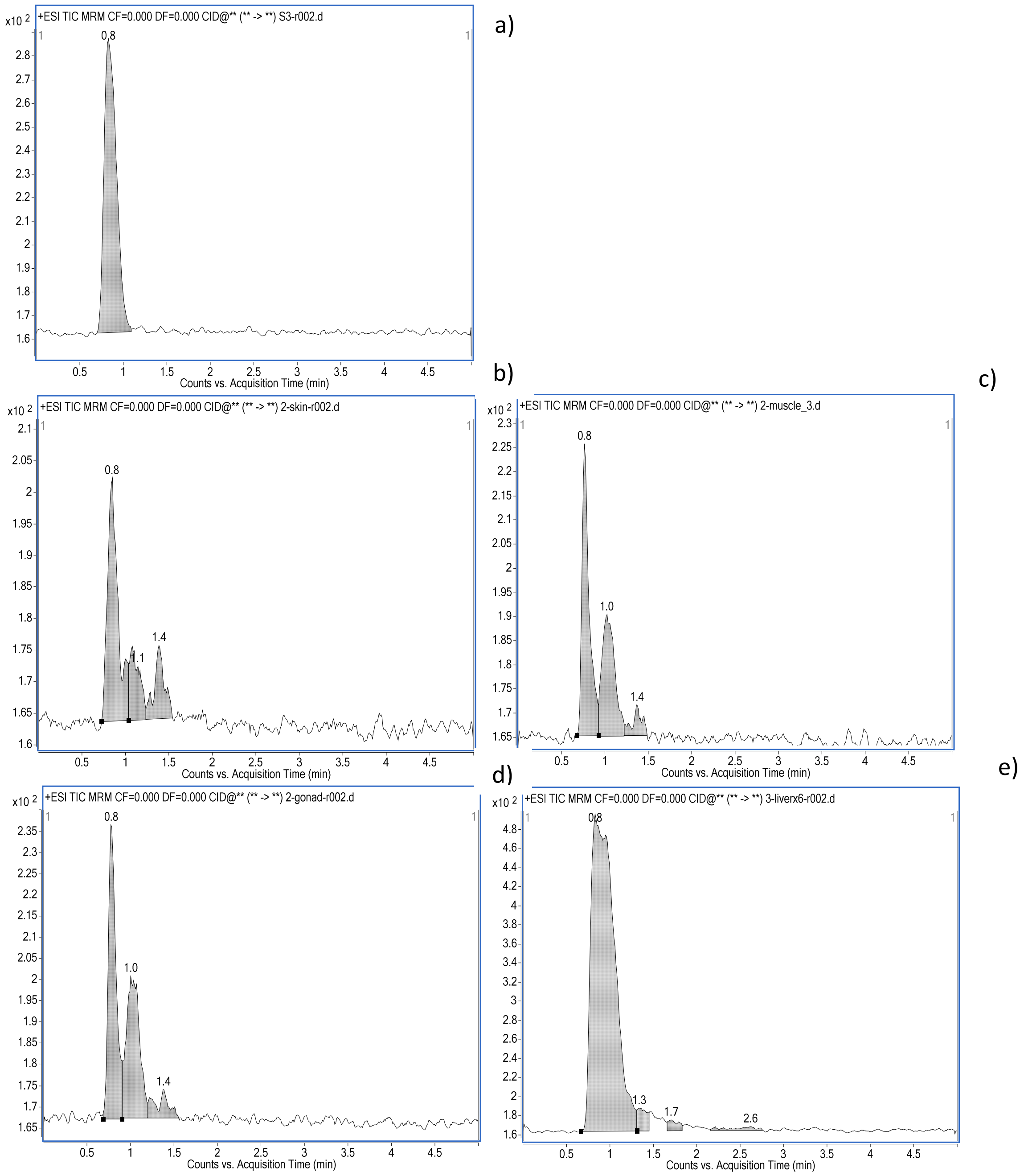

Figure S1. Total ion current plots of LC-MS/MS derived by the multiple reaction monitoring (MRM) of (a) TTX standard calibration solution (Retention Time = 0.8 min) and *Lagocephalus sceleratus* tissue extracts of: (b) skin, (c) muscle, (d) liver and (e) gonad, sampled at the South-Eastern Mediterranean Sea, Lebanon.

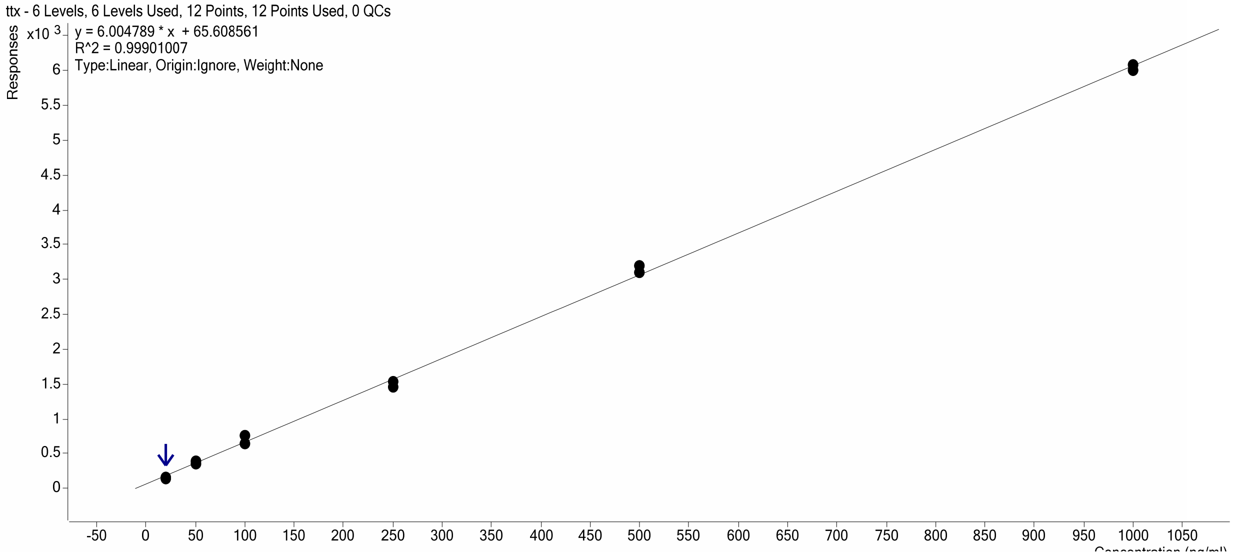

Figure S2. Response of the instrument and concentrations of standard solutions are linear, $r^2 > 0.99$, as shown for TTX.

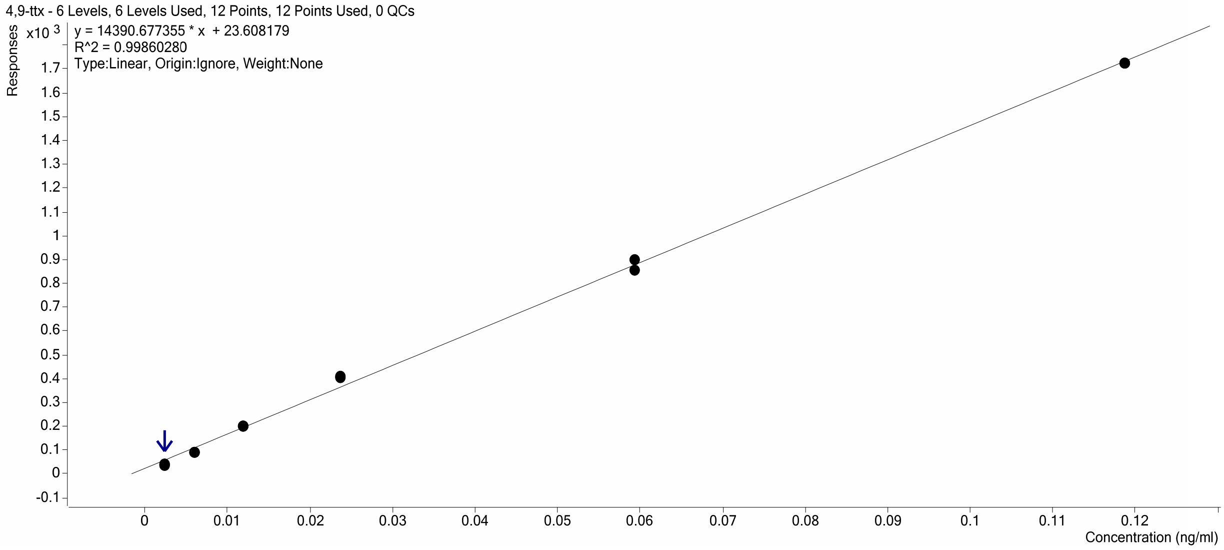

Figure S3. Response of the instrument and concentrations of standard solutions are linear, $r^2 > 0.99$, as shown for 4,9-anhydro TTX.
